# Palladium- and copper-mediated *N*-aryl bond formation reactions for the synthesis of biological active compounds

**DOI:** 10.3762/bjoc.7.10

**Published:** 2011-01-14

**Authors:** Carolin Fischer, Burkhard Koenig

**Affiliations:** 1 Institute of Organic Chemistry, University of Regensburg, Universitätsstr. 31, D-93053 Regensburg, Germany

**Keywords:** biologically active compounds, boronic acid, copper, N-arylation, palladium

## Abstract

N-Arylated aliphatic and aromatic amines are important substituents in many biologically active compounds. In the last few years, transition-metal-mediated *N*-aryl bond formation has become a standard procedure for the introduction of amines into aromatic systems. While N-arylation of simple aromatic halides by simple amines works with many of the described methods in high yield, the reactions may require detailed optimization if applied to the synthesis of complex molecules with additional functional groups, such as natural products or drugs. We discuss and compare in this review the three main N-arylation methods in their application to the synthesis of biologically active compounds: Palladium-catalysed Buchwald–Hartwig-type reactions, copper-mediated Ullmann-type and Chan–Lam-type N-arylation reactions. The discussed examples show that palladium-catalysed reactions are favoured for large-scale applications and tolerate sterically demanding substituents on the coupling partners better than Chan–Lam reactions. Chan–Lam N-arylations are particularly mild and do not require additional ligands, which facilitates the work-up. However, reaction times can be very long. Ullmann- and Buchwald–Hartwig-type methods have been used in intramolecular reactions, giving access to complex ring structures. All three N-arylation methods have specific advantages and disadvantages that should be considered when selecting the reaction conditions for a desired C–N bond formation in the course of a total synthesis or drug synthesis.

## Introduction

Palladium- and copper-mediated N-arylations are important tools in organic synthesis. Due to the widespread importance of aryl-*N* bond formation, many synthetic methods have emerged over the years. Besides the traditional Ullmann [1–2] and Goldberg [3–5] procedures, the palladium-catalysed reaction discovered by Buchwald [6–7] and Hartwig [8–9] has been a major breakthrough in this field. More recently, Chan [10] and Lam [11–12] introduced the copper-mediated arylation of N-nucleophiles using stoichiometric copper(II) acetate and boronic acids. Collman improved the procedure using catalytic amounts of [Cu(OH)TMEDA]_2_Cl_2_, omitting the base and working at room temperature [13–14]. Besides palladium and copper, nickel catalysis also allows the arylation of primary and secondary amines [15–16]. However, the three methods (Ullmann–Goldberg, Buchwald–Hartwig and Chan–Lam) have become standard procedures for *N*-aryl bond formation, and many examples illustrate their wide application in organic synthesis.

The chelating phosphines BINAP, DPPF [17] and D*t*BPF [18], commonly used for the Buchwald–Hartwig amination, were recently displaced by the biaryl-(dialkyl)phosphine or arylphosphinepyrrole ligands [18–20]. Industrial scale-up of these methods has already been applied on the 100 kg scale for arylpiperazines and different diarylamines [21]. In addition, Nolan et al. and Organ et al. have reported Pd-*N*-heterocyclic carbene (NHC)-catalysed Buchwald–Hartwig amination protocols that provide access to a range of hindered and functionalized aryl amines [22–24]. Aryl bromides are most frequently applied as substrates for the coupling of primary and cyclic secondary amines [17]. In the presence of a weak base such as caesium carbonate, many functional groups are tolerated, while NaO*t*-Bu has limitations when base-labile functional groups are present. Electron-neutral and electron-poor aryl bromides are suitable substrates [17], and *ortho*-substituents on the aryl halide are tolerated. In contrast, electron-rich aryl bromides give only poor results. Recently, the modular synthesis of indoles by a palladium-catalysed cascade process provided an efficient entry to substituted indoles [25].

Although copper is less toxic and less expensive than palladium, the required harsh conditions, the limited range of suitable substrates and moderate yields prevented the use of Ullmann-type reaction from reaching its full potential for a long time. Aryl halides activated by electron-withdrawing groups can only be converted at high temperatures (210 °C) using stoichiometric amounts of copper. The discovery of efficient copper/ligand systems enabled the use of catalytic amounts of metal under milder conditions (90–100 °C) and resulted in good yields [4,26]. Copper-diamine-catalysed N-arylation facilitated the arylation of pyrroles, pyrazoles, indazoles, imidazoles, triazoles, benzimidazoles and indoles [27–29]. Besides aryl halides as the aryl donor, arylsiloxanes [30], arylstannanes [31], iodonium salts [32], aryl lead(IV) triacetates [33] and pentavalent organobismuth reagents [34] have also been used as aryl donors for copper-mediated C–N couplings.

Further improvement of N-arylation conditions was achieved by the use of arylboronic acids. The reagents are not sensitive to air; the reaction proceeds at room temperature [35–36] and in aqueous solution [37]. However, the reactions are very slow and require several hours or even days for completion [38].

In general, there are a wide variety of protocols describing the metal-mediated arylation of amines [17,37,39], amides [38], imides [38], imidazoles [14,37,40], benzimidazoles [40–41], sulfonamides [38], pyrroles [42] and lactams [43]. The three typical methods for N-arylation have been extensively reviewed concerning scope and limitation of these reactions [4,44–48].

However, the application of palladium- and copper-mediated N-arylation reactions in the synthesis of complex molecules such as natural products or drugs is, in comparison to standard small-molecule N-arylation, not always straightforward and requires specially optimized conditions. Since amine- and amide-substituted aromatics and heteroaromatics are typical structures in medicinal chemistry and natural product synthesis, a broad application of catalytic C–N-arylation is highly desirable.

Evano et al. recently reviewed copper-mediated C–N-arylation reactions in natural product syntheses and discussed different examples from total synthesis using the arylation of alkylamines, amides, carbamates, *N*-heterocycles, enamines and intramolecular N-arylation reactions [45].

We compare here the success of the different C–N-arylation reactions as applied to the synthesis of more complex structures and discuss selected examples of palladium- and copper-mediated reactions for the synthesis of bioactive compounds in terms of scope and limitation. If available, we directly compare the different methods and advantages of specific reaction conditions. The review should help synthetic chemists to select the most suitable catalytic C–N-arylation method for their target molecule.

## Review

### Pd-catalysed synthesis of biological active molecules

**Scheme 1:**
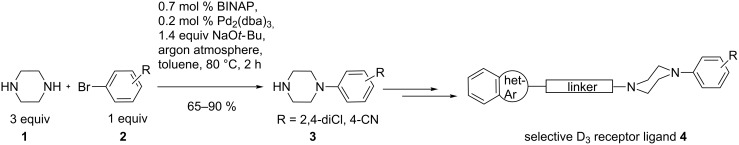
Synthesis of selective D_3_ receptor ligands.

A typical example for the application of palladium-catalysed *N*-aryl bond formation is the synthesis of highly selective D_3_ receptor ligands **4**. Piperazine (**1**) and a substituted aryl bromide **2** (Scheme 1) are coupled in the initial step of the synthesis.

The electron-withdrawing substituents (nitrile and chloro) on the aryl bromide assisted the reaction, and reported yields of **3** are in the range of 65–90%. This method represents conditions from early catalyst generations and allowed the synthesis of a library of 18 compounds, which were investigated to identify a possible structure–activity relationship [49].

**Scheme 2:**
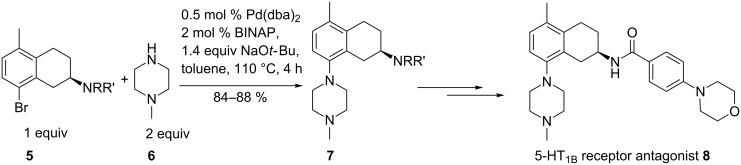
Synthesis of a novel 5-HT_1B_ receptor antagonist.

Federsel et al. used a piperazine derivative **6** and an aryl halide **5** for the preparation of a CNS-active substituted chiral aminotetralin **7** (Scheme 2) [50]. The 5-HT_1B_ receptor antagonist **8** was developed for the treatment of certain neuronal disorders. Different syntheses have been developed in which Pd(OAc)_2_ proved to be the best catalyst in comparison to the dba complex, and BINAP the best ligand. There was no difference if the racemic or the enantiomeric pure ligand was used. The use of Pd(OAc)_2_ is advantageous on a larger scale due to low cost and easy handling. The choice of base was crucial. NaOMe and NaOEt gave only low conversions in comparison to NaO*t*-Bu. The reaction tolerated up to 0.06% water. Thus, the conditions could be optimized for the final use in a pilot plant in which batches of 125 kg were synthesized in a robust and reproducible manner [51].

**Scheme 3:**
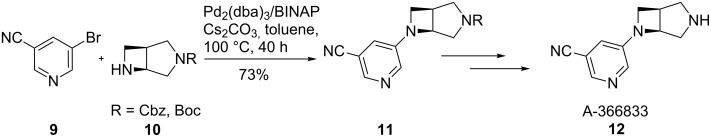
Synthesis of A-366833, a selective α4β2 neural nicotinic receptor agonist.

Compound A-366833 (**12**) was found to be a broad-spectrum analgesic having an improved safety profile relative to other pyridine-containing analgesics. The efficient synthesis of this compound used a palladium-catalysed coupling for the final step connecting 3-bromo-5-cyanopyridine (**9**) with the protected (1*R*,5*S*)-3,6-diazabicyclo[3.2.0]heptane **10**. The use of Cs_2_CO_3_ instead of the conventional base *t*-BuONa increased the yield of **11** from 47% to 73% (Scheme 3) [52]. The enantioselective synthesis of A-366833 allowed large-scale preparation required for preclinical investigations.

**Scheme 4:**
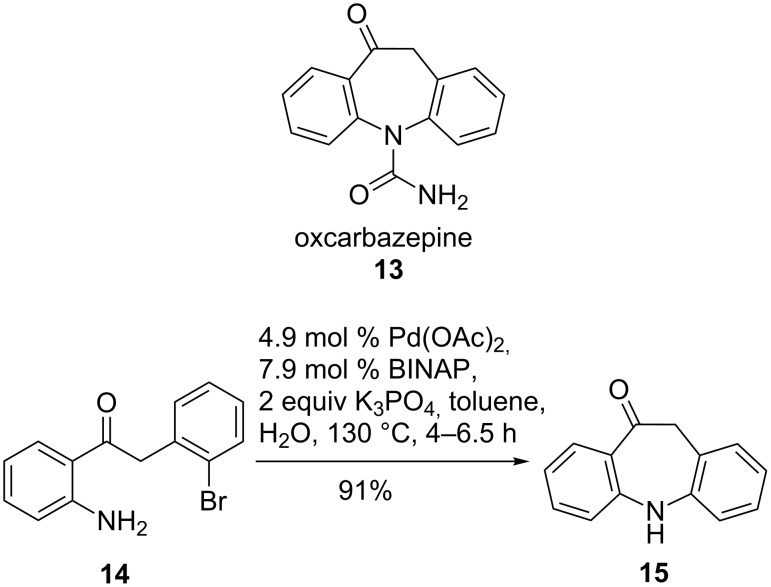
A new route to oxcarbazepine.

Oxcarbazepine (**13**, Trileptal) is one of the most prescribed drugs for the treatment of epilepsy due to improved tolerability profile compared to carbamazepine. Additionally, its analgesic properties and successful treatment of mood disorders and mania make it an important therapeutic agent. In 2005, a new method that involved a cyclization step by intramolecular N-arylation of **14** was developed that led to improved access to compound **15** due to better availability of the starting material and a simple palladium source. Under optimized conditions, palladium catalysis yielded the tricyclic skeleton **15** in 91% and reduced the amount of dehalogenated by-product to 4% (Scheme 4). The beneficial effect of added water may be due to better dissolution of K_3_PO_4_. Scale-up to gram amounts was possible without any significant decrease in yield. The reaction failed with both copper-mediated Ullmann-type reactions and heterogeneous palladium catalysis [53].

Intramolecular palladium-catalysed N-arylations have been applied to substituted arenes and thiophenes with good to excellent yields. Electron-rich bromides gave the best results, while pyridine derivatives were unreactive [54].

Another intramolecular approach enabled the stereoselective synthesis of an atropisomeric *N*-(2-*tert*-butylphenyl)lactam as an intermediate for norepinephrine transporter (NET) inhibitor **16**. NET inhibitors were developed to treat a variety of mental disorders such as depression and attention deficit hyperactivity disorder (ADHD).

**Scheme 5:**
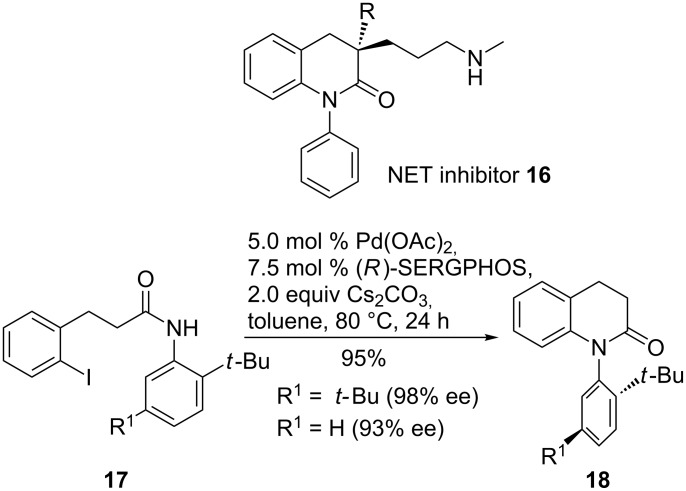
Synthesis of key intermediates for norepinephrine transporter (NET) inhibitors.

Screening different ligands, SERGPHOS appeared to give the highest stereoselectivity of **18** for the N-arylation of **17** (Scheme 5) [55]. Other conditions were not investigated in this case.

**Scheme 6:**
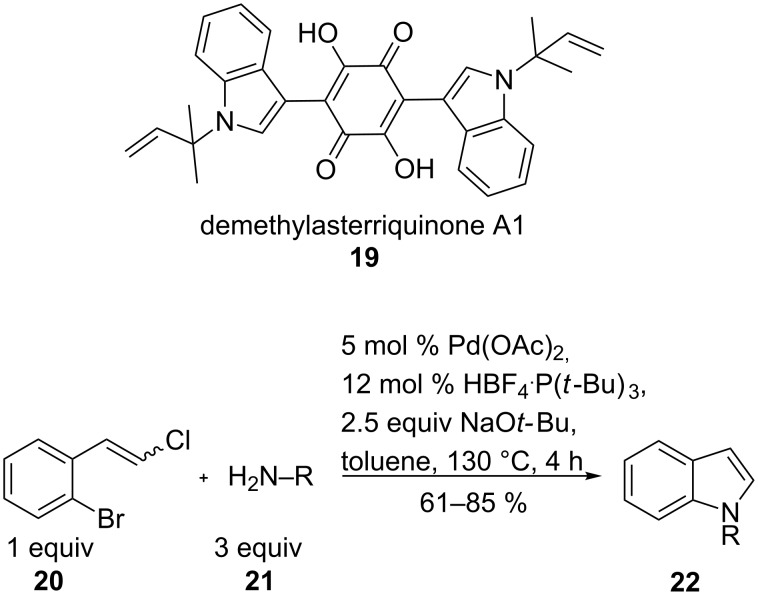
N-Annulation yielding substituted indole for the synthesis of demethylasterriquinone A1.

Another example for BINAP not being the favoured ligand is the synthesis of the fungal natural product demethylasterriquinone A1 (**19**). Asterriquinones show different biological functions including anti-tumour activity and are used as insulin mimetics. The palladium-catalysed coupling of styrene **20** with sterically demanding N-nucleophiles **21** gave the indole building blocks **22** for the natural product synthesis (Scheme 6). In this case, P(*t*-Bu)_3_ was the appropriate ligand. As starting materials cyclic and aromatic amino compounds as well as substituted styrenes were tolerated (yields 61–85%). Only nitro-substituted styrene resulted in poor conversion (34%) [56].

**Scheme 7:**
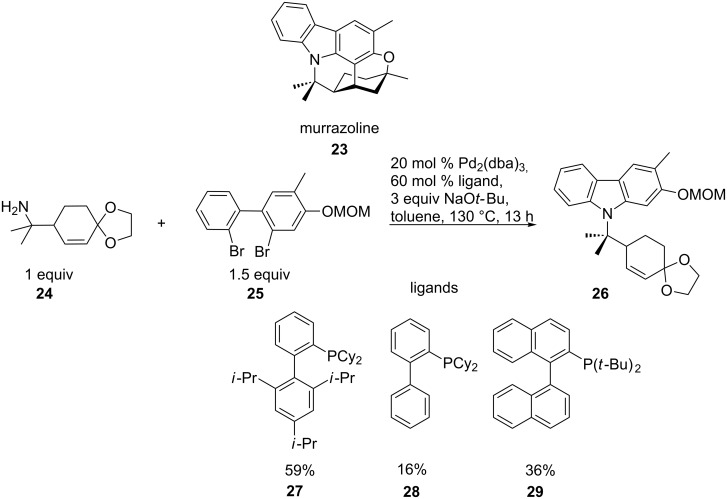
Palladium-catalysed double N-arylation contributing to the synthesis of murrazoline.

The synthesis of the natural product murrazoline is a further example of connecting a sterically demanding N-nucleophile at two positions. Murrazoline (**23**), a carbazole alkaloid isolated from the shrub *Murraya*, is used in folk medicine for the treatment of eczema, rheumatism and dropsy, as an analgesic, and in anaesthesia. It is known to be a potent platelet aggregation inhibitor. The double N-arylation of **25** was carried out under standard conditions using **24**, and the best yield of **26** was achieved with the dicyclohexyl(2',4',6'-triisopropylbiphenyl-2-yl)phosphine ligand **27** (59%, Scheme 7) compared to the ligands, **28** and **29** [57].

**Scheme 8:**
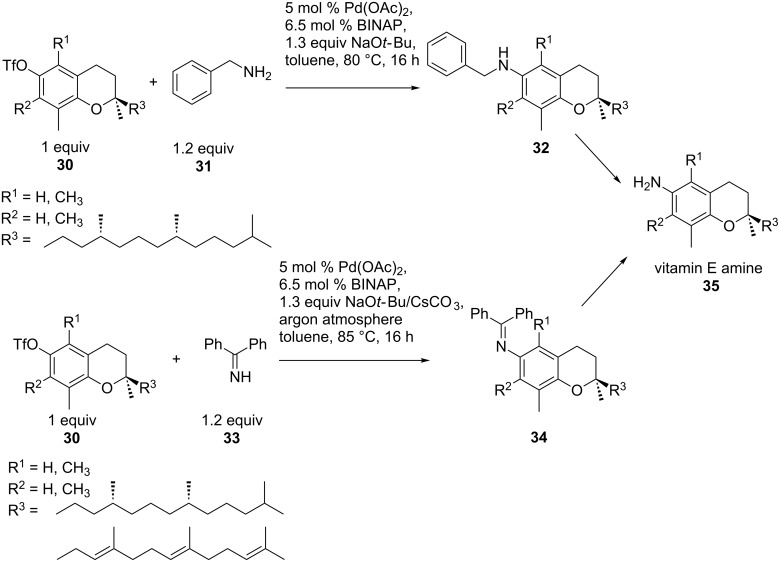
Synthesis of vitamin E amines.

The synthesis of vitamin E amines **35**, possessing anti-proliferative activity, could be realized by the easy and efficient procedure of coupling **30** to **31** or **33** according to Scheme 8. Enantiopure tocopheramines **32** and tocotrienamines **34** were synthesized using *N*-aryl amination as key the step. Here, the leaving group was triflate instead of the more commonly used halides and yields ranged from 10% to 80% [58].

**Scheme 9:**
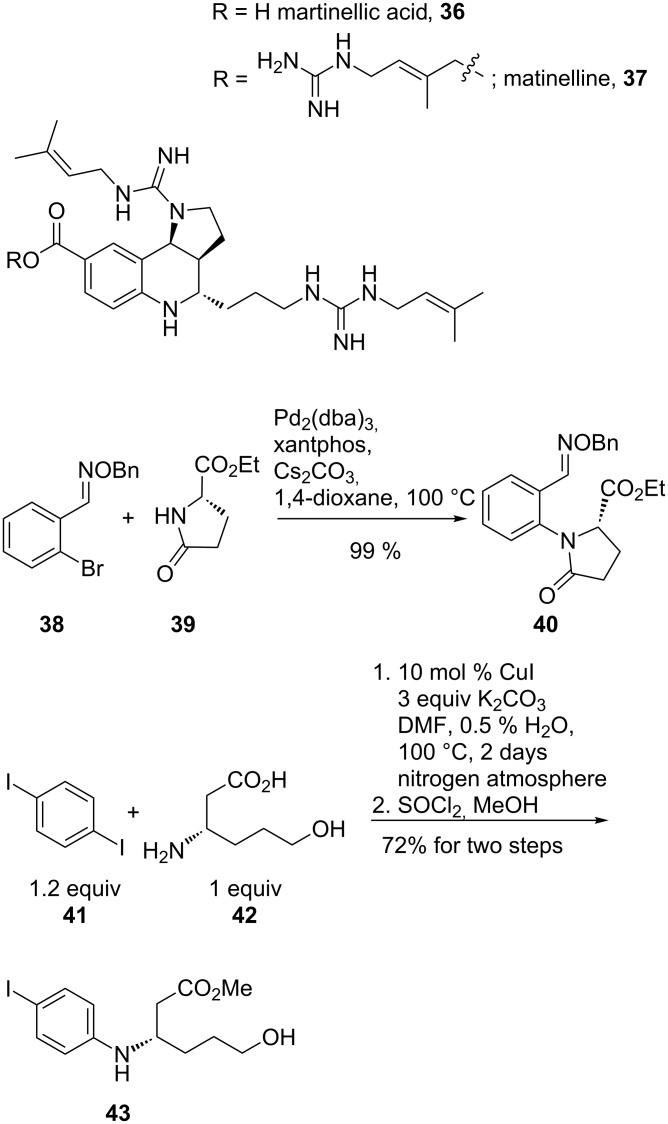
Improved synthesis of martinellic acid.

An example illustrating both methods, i.e., the palladium- and the copper-catalysed N-arylation, is the synthesis of the natural product martinellic acid (**36**) and one of its derivatives (Scheme 9). The two alkaloids, martinellic acid (**36**) and martinelline (**37**), possess antagonist activity towards bradykinin B1 and B2 receptors. The key step of the synthetic sequence is the connection of an amino acid derivative to the aromatic core.

Miyata et al. reported a nearly quantitative yield of **40** (99%) for the palladium strategy in the reaction between (*S*)-ethyl 5-oxopyrrolidine-2-carboxylate (**39**) and 2-bromobenzaldehyde *O*-benzyl oxime (**38**), and only a moderate yield (53%) using CuI [59]. Targeting the same product, Ma et al. used copper catalysis some years previously. The coupling of 1,4-diiodobenzene (**41**) and (*S*)-ethyl 3-amino-6-hydroxyhexanoate (**42**), and subsequent esterification resulted in an overall yield of 72% for the two steps to **43** [60–61]. The specific yield for the copper-catalysed reaction step was not reported.

### Cu-catalysed synthesis of biological active molecules

Copper-catalysed N-arylation reactions were applied to introduce diversity into new ABCB1 transporter modulators. The tariquidar (**44**) derived compounds showed log P-dependent inhibition activity of the ABCB1 transporter, which represents an important component of the blood–brain barrier and is a major limitation in cancer chemotherapy.

**Scheme 10:**
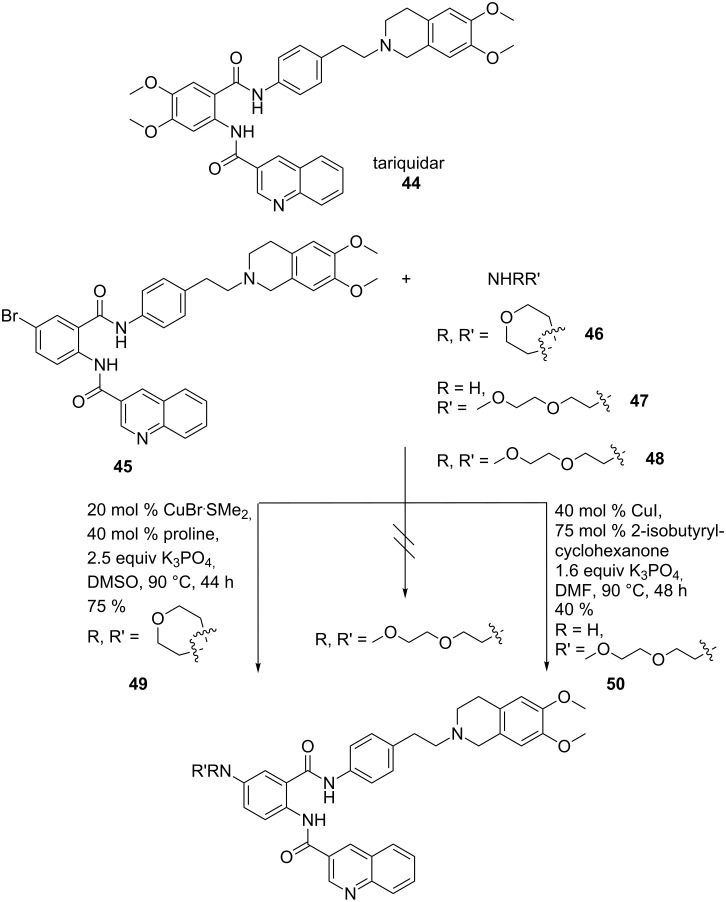
New tariquidar-derived ABCB1 inhibitors.

Primary and cyclic secondary amines were coupled to **45** to give moderate to good yields of **49** and **50**. Secondary acyclic amines **48** were unreactive: A variety of different conditions were investigated including the variation of base, ligand and copper source. Scheme 10 shows the optimized conditions for coupling with morpholine (**46**) and 2-(2-methoxyethoxy)ethanamine (**47**). Palladium-catalysed reactions failed due to the low solubility of the bromo compound in toluene [62].

**Scheme 11:**
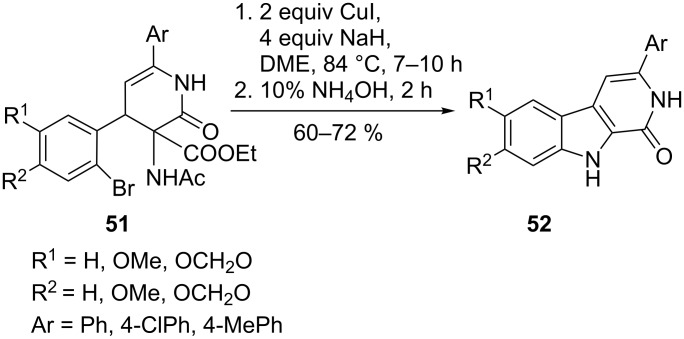
β-Carbolin-1-ones as inhibitors of tumour cell proliferation.

Another class of compounds used in anti-tumour treatment are β-carbolin-1-ones **52** due to their inhibition of cell proliferation. These compounds were accessible via intramolecular coupling between an aryl bromide and an acylated amine **51**). In comparison to the Goldberg reaction conditions (CuI, NaH, DMF at 90 °C for 2 h), the change of the solvent to DME was crucial a gave good yields of up to 72% (Scheme 11). Additionally, the reaction was sensitive to the amount of NaH employed. Substituents on the aryl bromide affected the yield only marginally [63].

**Scheme 12:**
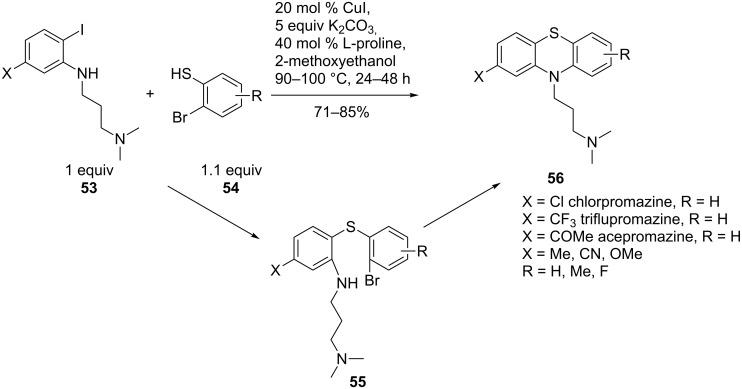
Copper-catalysed synthesis of promazine drugs.

**Scheme 13:**
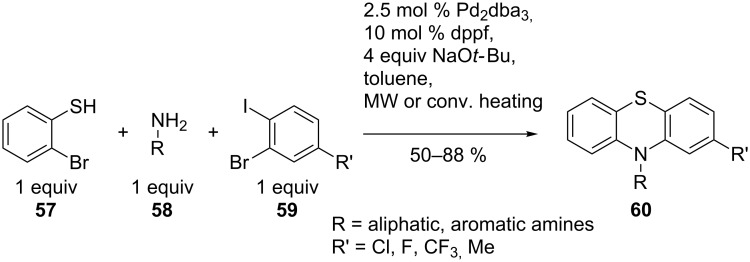
Palladium-catalysed multicomponent reaction for the synthesis of promazine drugs.

A second example of intramolecular coupling is the synthesis of promazine drugs (**56**) that are interesting due to their clinical use for psychotropic medication. The CuI/L-proline-catalysed cascade process, developed by Ma et al., gave the best yields when 2-methoxyethanol was used as solvent, compared to DMSO, dioxane etc. The reaction conditions tolerated both electron-rich and electron-deficient substituents on the aniline **53** and on the 2-bromobenzenethiol **54** in different substitution patterns. This reaction is thought to proceed via intermediate **55**. The catalytic synthetic sequence is an inexpensive and efficient route to the target compounds (Scheme 12) [64]. A three-component coupling reaction of 2-bromobenzenethiol **57**, a primary amine **58** and 1-bromo-2-iodobenzenes **59**, also targeting promazine derivatives **60**, was successfully under palladium catalysis (Scheme 13). The method benefits from controlled regiochemistry and is applicable to various aliphatic and aromatic amines **58**. Although the reaction scope was limited to 1-bromo-2-iodobenzenes **59**, scale-up to multigram quantities was possible [65].

**Scheme 14:**
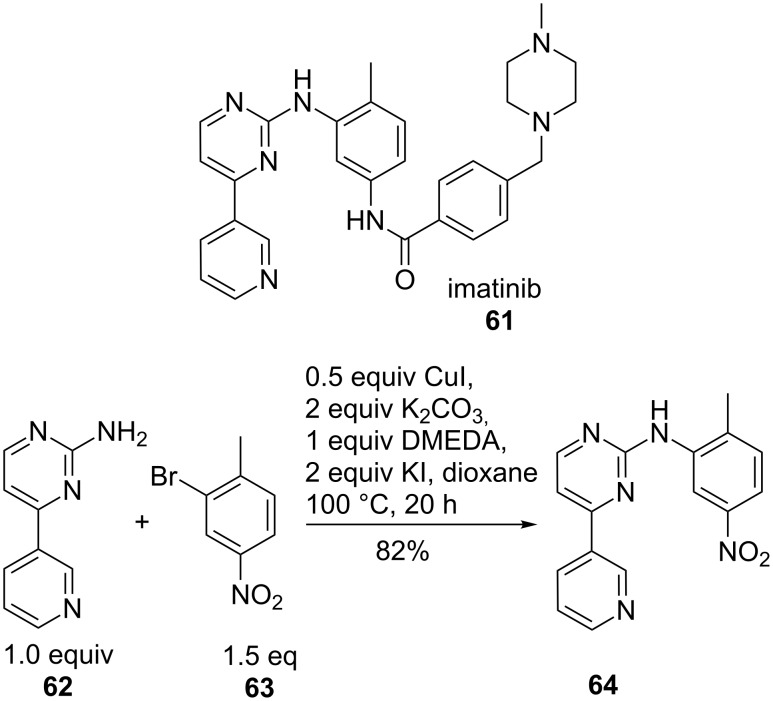
Key intermediate for imatinib.

A key intermediate **64** of imatinib **61**, a standard anti-cancer drug for the treatment of chronic myelogenous leukaemia and gastrointestinal tumours, was prepared in 82% yield via copper-mediated N-arylation of **62** and **63** (Scheme 14). By screening different reaction conditions, Cu(I) was found to be the best copper source compared to Cu(0) and Cu(II). In the series of aryl halides, aryl iodides **63** were the most active. The presence of air and water gave only slightly lower yields. Other heteroarylamines also gave good yields [39].

**Scheme 15:**
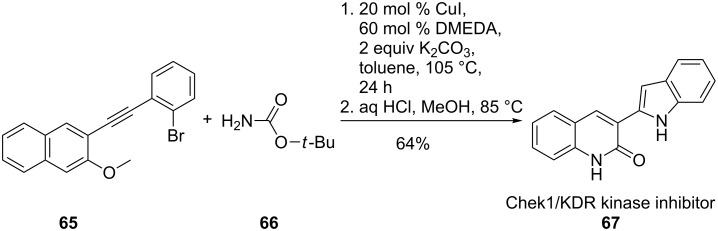
Synthesis of an effective Chek1/KDR kinase inhibitor.

The copper-catalysed domino-indole synthesis facilitated access to new Chek1/KDR kinase inhibitors **67** from phenylethynylnaphthalenes **65** and *tert*-butyl carbamate (**66**) (Scheme 15). The broad scope of this method improved the synthesis of diversely substituted indoles. In addition, *N–*H, *N*-acyl and *N*-aryl indoles were accessible. In this case, DMEDA turned out to be the crucial ligand [66].

**Scheme 16:**
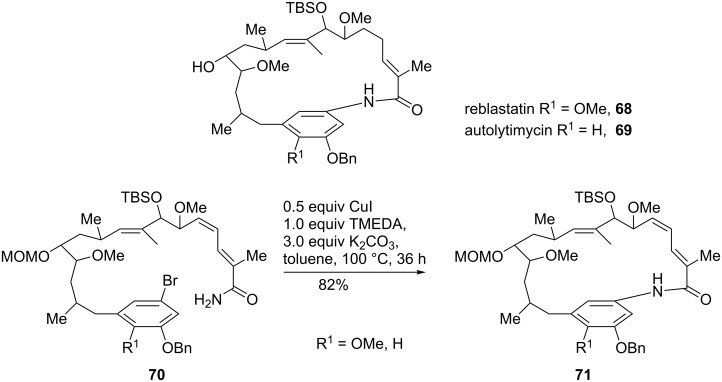
Macrocyclization as final step of the synthesis of heat shock protein inhibitor.

Examples for the synthesis of complex biologically active compounds are reblastatin (**68**) and autolytimycin (**69**). These potent inhibitors of heat shock protein 90, an important therapeutic target for cancer treatment, were accessed by a copper-mediated macrocyclization step of **70** to **71** in high yield (82%, Scheme 16) [67]. The same method was employed in the total synthesis of geldanamycin and other 8- to 14-membered lactam-containing natural products [68].

**Scheme 17:**
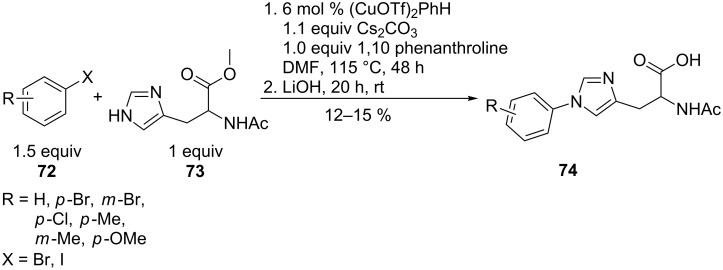
Synthesis of *N*-arylimidazoles.

A number of biological targets, such as blocking cytochrome C oxidase, have made *N*-arylhistidines **74** important drug candidates. The quite simple-looking copper triflate-catalysed conversion of aryl halides **72** with the *N*-acylhistidine **73** was total regioselective on the imidazole ring, but suffered from complicated isolation procedures due to difficulties in separating the product from stoichiometric quantities of the ligand. Sufficient reactivity was only observed for aryl iodides and low overall yields were observed including at the subsequent ester cleavage step (Scheme 17) [69].

**Scheme 18:**
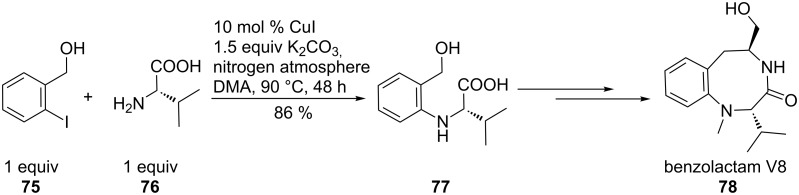
Synthesis of benzolactam V8.

**Scheme 19:**
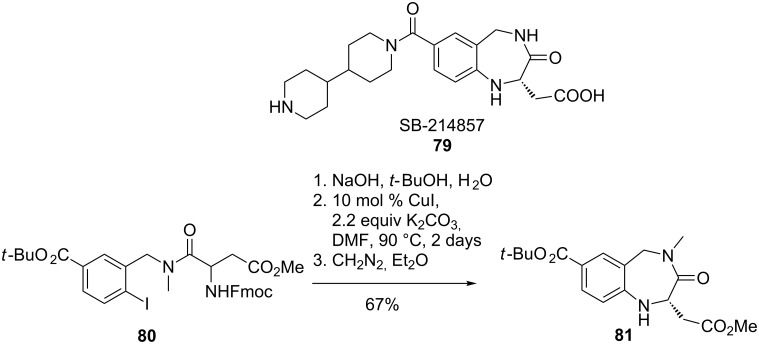
Synthesis of an intermediate for lotrafiban (SB-214857).

In the well-known synthesis of benzolactam V8 (**78**) [70], Ma et al. proved the accelerating effect of α- (**76**) and β-amino acids (**80**) on aryl aminations (Scheme 18 and Scheme 19). Lotrafiban (SB-214857, **79**), a potent GPIIb/IIIa receptor antagonist inhibiting platelet aggregation, was efficiently obtained by an Ullmann-type aryl amination reaction. Despite the disadvantage of long reaction time (2 days), the intermediate **81** was obtained in enantiopure form [71].

**Scheme 20:**
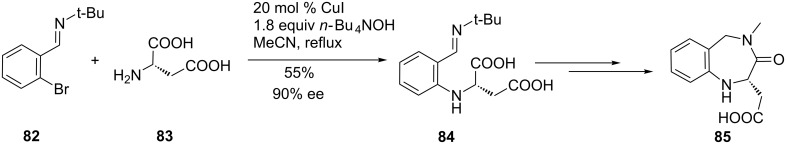
Intermolecular effort towards lotrafiban.

The very similar intermediate **85**, published at the same time, was synthesized by the coupling of L-aspartic acid (**83**) to aryl bromide **82** in 55% yield and 90% ee (Scheme 20). Under these reaction conditions, CuI was found to be the copper source that caused the least amount of racemization [72].

### Chan–Lam arylation in the synthesis of biologically active molecules

**Scheme 21:**
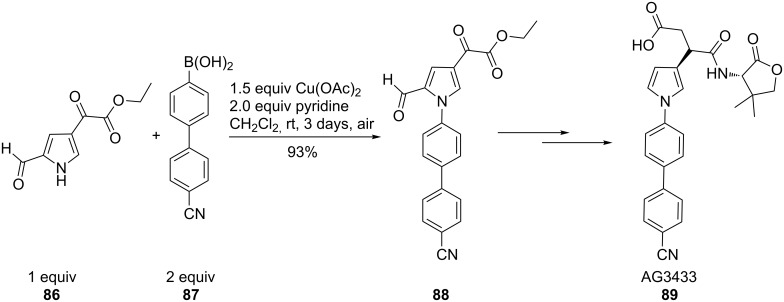
Synthesis of matrix metalloproteases (MMPs) inhibitor.

A key intermediate (**88**) for the potent matrix metalloproteases (MMPs) inhibitor AG3433 (**89**) was synthesized by coupling an electron-deficient pyrrole (**86**) with an arylboronic acid (**87**) in excellent yield (93%, Scheme 21). Screening numerous boronic acids it was found that only boronic acids containing electron-donating or weakly electron-withdrawing substituents were suitable. Pyrroles lacking a substituent in the 2-position, which is supposed to support the reaction by a chelating effect with the copper ion, did not succeed in the coupling reaction. Further disadvantages were the required stoichiometric amount of copper and the long reaction time of 3 days [42].

**Scheme 22:**
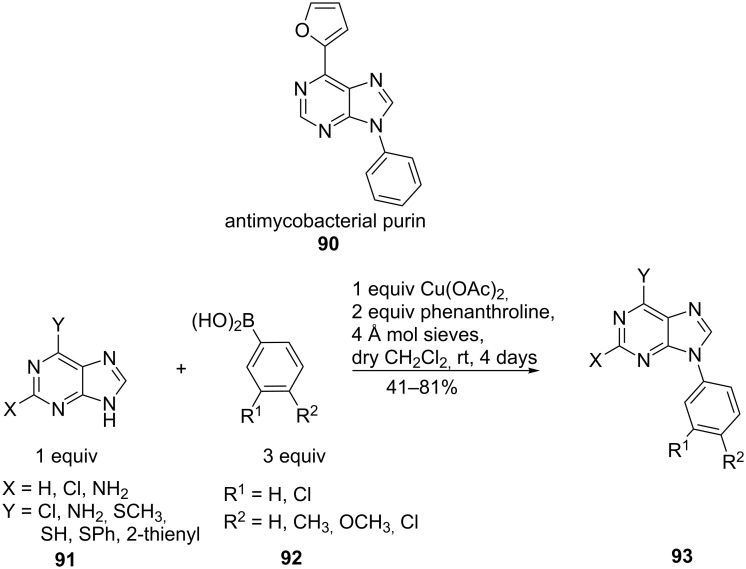
Regioselective 9-N-arylation of purines.

For detailed insight into the structural requirements of active antimycobacterial purines **90**, an easy access to 9-*N*-arylpurines **93** was required. Complete regioselectivity, and in most cases high chemoselectivity, was achieved by reacting 9-*N*-purines **91** with an excess of arylboronic acid **92** in the presence of copper(II) acetate, molecular sieves and phenanthroline (Scheme 22). Bakkestuen and Gundersen showed that electron-donating and electron-withdrawing substituents on the arylboronic acid were tolerated. However, adenine was unreactive under these conditions, probably due to its low solubility [73].

**Scheme 23:**
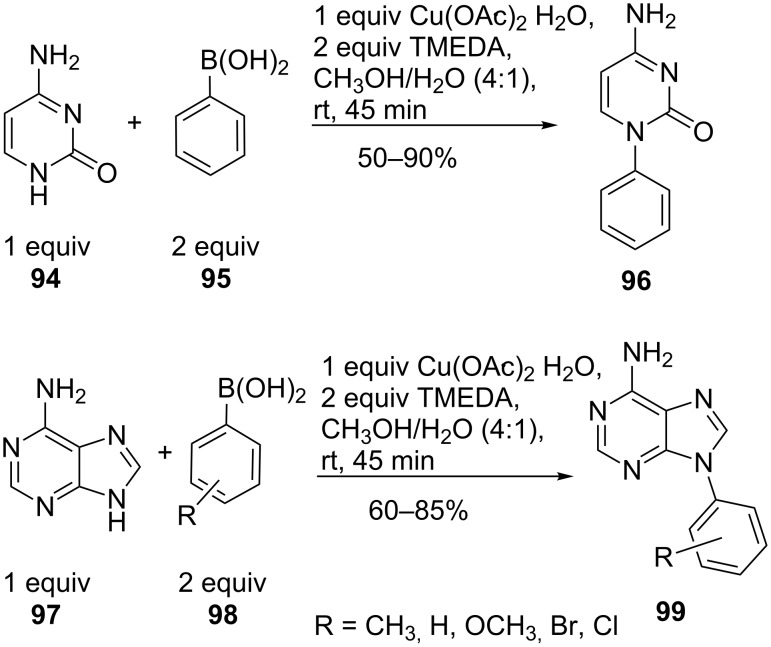
N-Arylation of adenine and cytosine.

Slight changes of the procedure, the use of a protic solvent, enabled the conversion of adenine (**97**) and cytosine (**94**) (Scheme 23). These conditions tolerated both electron-donating and electron-withdrawing substituents at the *o*-, *m*-, *p*-positions in the phenylboronic acid (**98**) and resulted in moderate to excellent yields [74].

**Scheme 24:**
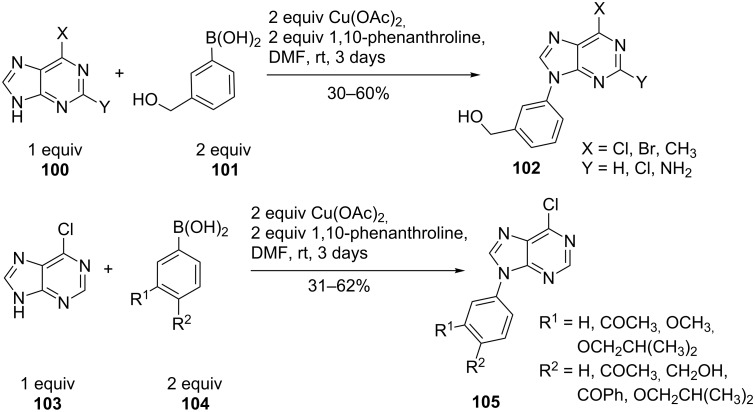
9-*N*-Arylpurines as enterovirus inhibitors.

9-*N*-Arylpurines **102** and **105** are used as a new class of inhibitors against enteroviruses, which are responsible for a variety of acute human diseases such as respiratory infections, meningitis, pancreatitis and others. The conditions reported by Bakkestuen and Gundersen [73] gave only a low yield (26%). DMF as solvent and the absence of molecular sieves improved the yield (Scheme 24). The reaction conditions were compatible for different purine bases (**100**, **103**) and for a variety of functional groups in the arylboronic acid (**101**, **104**). Thus, a single reaction step from commercial precursors allowed the synthesis of new enterovirus inhibitors with activity in the low µM range [75].

**Scheme 25:**
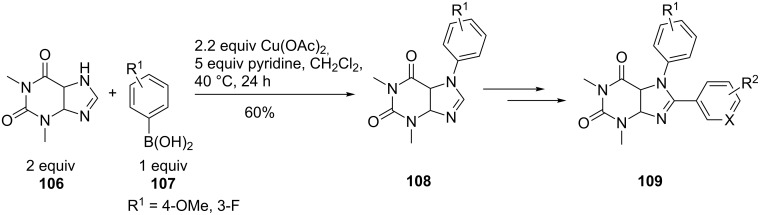
Xanthine analogues as kinase inhibitors.

A very recent example of 7-N-arylation of purines is the synthesis of highly substituted xanthine derivatives **109** as fluorescent and potent kinase inhibitors. The conditions of Bakkestuen et al. were modified and gave yields of up to 60% for the optimized conditions with pyridine as the base and heating the reaction mixture to 40 °C for 24 h (Scheme 25). In addition to the *p*-methoxy substituent in the boronic acid **107**, *p*-methyl and *m*-fluoro substituents were also tolerated. The xanthine compounds showed good anti-proliferative activity and exhibited a significant fluorescence response [76].

**Scheme 26:**
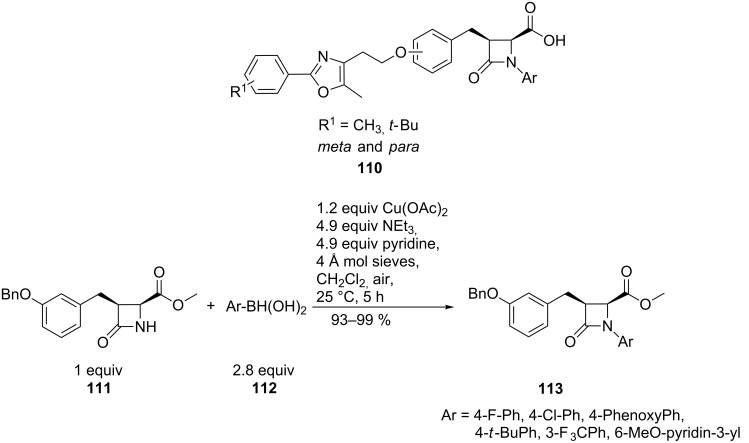
Synthesis of dual PPARα/γ agonists.

The peroxisome proliferator-activated receptors (PPARs), members of the nuclear hormone receptor superfamily, are important targets in the treatment of diabetes and dyslipidemia. Azetidinone acid derivatives **110** were discovered to be new subtype-selective PPARα/γ agonists. For detailed structure–activity relationship (SAR) studies, diversity was introduced very efficiently by a copper-mediated N-arylation of azetidinones **111** with different arylboronic acids **112** to give **113** in nearly quantitative yields (Scheme 26) [77]. The palladium-catalysed N-arylation of 2-azetidinones was only previously described for unsubstituted azetidinones [78].

**Scheme 27:**
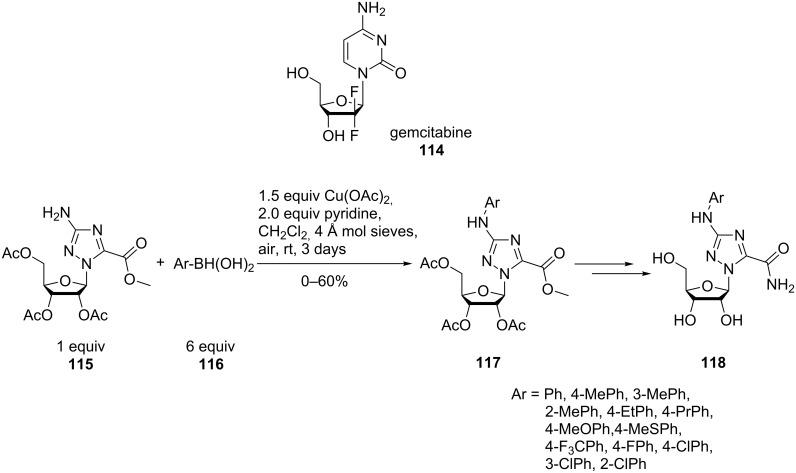
*N*-Aryltriazole ribonucleosides with anti-proliferative activity.

*N*-Aryltriazole ribonucleosides **118** with potent anti-proliferative activity against drug-resistant pancreatic cancer were prepared by coupling 3-aminotriazole with various boronic acids. Similar to the method developed for *N*-aryltriazole acylonucleoside analogues [79], N-arylation was performed in the presence of slightly greater than a stoichiometric amount of Cu(OAc)_2_, pyridine and freshly activated molecular sieves in CH_2_Cl_2_ at room temperature and the open air for 3 days (Scheme 27). There was no clear trend as to the effect of electron-donating or electron-withdrawing substituents of the arylboronic acid **116** on the course of the reaction. However, sterically hindered, *ortho*-substituted arylboronic reagents were unreactive. Although the coupling resulted only in moderate yields, a new anti-cancer drug candidate with improved potency on human pancreatic cancer cells, compared to gemcitabine (**114**), was obtained [80].

## Conclusion

Palladium- and copper-mediated N-arylations have been shown to be powerful methods for introducing aryl and heteroaryl substituents. However, for substrates with more complex structures, the performance and applicability of the different reactions are often difficult to predict. The three reaction types, palladium-catalysed and copper-catalysed N-arylations of aryl halides, and copper-catalysed N-arylations using boronic acids show some distinct differences. Palladium-catalysed N-arylation reactions typically require reaction temperatures of 80–130 °C and prolonged reaction times up to 40 h. In most of the cases, the reactions must be conducted under an inert atmosphere; toluene is a favourite solvent and BINAP a commonly used ligand. Strong bases are required and water is added to dissolve them. Substituents in the starting material, which are base labile are not tolerated. With low catalyst loading and good availability of the palladium sources, reactions on a larger scale are possible. Sterically demanding N-nucleophiles, as well as cyclic and aromatic amino compounds, are suitable coupling partners with aryl bromides.

In contrast to palladium catalysis, Ullmann-type coupling reactions tolerate atmospheric oxygen. However, reaction temperatures (90–115 °C) and reaction times (48 h) are comparable to the palladium-catalysed processes. Ligand-free and ligand-assisted reaction conditions have been applied in the synthesis of biologically active compounds. DMEDA, proline and phenanthroline are the most commonly used ligands. In some cases, the up to stoichiometric amount of ligand made work-up procedures difficult. In general, CuI is the most efficient catalyst. The reaction conditions are applicable to primary, cyclic secondary aliphatic amines, electron-rich and electron-poor anilines, and heteroarylamines. Macrocyclization using amides and aryl bromides is possible. Both palladium-catalysed and Ullmann-type N-arylation reactions were successfully applied for both inter- and intramolecular reactions. An advantage of the Ullmann-type reactions is the lower cost of catalyst metal salts and ligands.

The mildest conditions for N-arylation reactions are provided by the Chan–Lam arylation procedure, but conversion at room temperature requires long reaction times of up to 4 days. The general absence of ligands is an additional advantage facilitating product purification. However, 1–2 equiv of Cu(OAc)_2_ and large excess of boronic acid have to be used. Boronic acid reagents can have electron-donating and electron-withdrawing substituents in *ortho*-, *meta*- and *para*-positions, but boronic acids with sterically demanding substituents were found to be unreactive. Many precursors for the starting materials used in Chan–Lam arylations are commercially available.

None of the three reactions is clearly superior as method for all N-arylations. Depending on the substrate and the summarized constraints, the preferred method must be selected. In many cases optimization or adaptation of the standard protocols to the specific substrate is required. Nevertheless, the modern N-arylation methods have become an indispensable tool in organic chemistry and significantly facilitate the synthesis of complex natural products and drugs.
